# Phylogenomic characterization of *Flavobacterium psychrophilum* isolates retrieved from Turkish rainbow trout farms

**DOI:** 10.1111/jfd.13961

**Published:** 2024-05-22

**Authors:** Izzet Burcin Saticioglu, Muhammed Duman, Nihed Ajmi, Soner Altun, Tatiana Rochat, Eric Duchaud

**Affiliations:** ^1^ Department of Aquatic Animal Disease, Faculty of Veterinary Medicine Bursa Uludag University Bursa Turkey; ^2^ Graduate School of Health Sciences Bursa Uludag University Bursa Turkey; ^3^ Université Paris‐Saclay, INRAE, UVSQ, VIM Jouy‐en‐Josas France

**Keywords:** aquaculture, BCWD, MLST, phylogenomic analysis, RTFS

## Abstract

*Flavobacterium psychrophilum*, a devastating fish pathogen, is responsible for bacterial cold‐water disease (BCWD), also known as rainbow trout fry syndrome. *F. psychrophilum* is the main causative agent of outbreaks in rainbow trout farms, especially at early live stages. In the present study, we aimed to characterize *F. psychrophilum* Turkish isolates. Eighteen isolates were retrieved from BCWD outbreaks between 2014 and 2021. In vitro phenotypic characterization showed gelatin and casein hydrolysis capacities and in vitro adhesion for all isolates, whereas elastinolytic activity was present for 16 of 18 isolates. We used complete genome sequencing to infer MLST‐type, serotype and phylogenetic reconstruction. Strikingly, one strain isolated from Coruh trout (FP‐369) belongs to ST393, a previously undescribed ST, and is phylogenetically distant from the other isolates. However, all strains retrieved from rainbow trout belong to the well‐characterized clonal complex CC‐ST10, 12 of 17 were tightly connected in a single cluster. Several serotypes (Types ‐1, ‐2 and ‐3) were represented among isolates, but no correlation was observed with geographic origins. This analysis suggests a regional dissemination of an epidemic, disease‐producing bacterial population. This study provides a basis for epidemiological surveillance of isolates circulating in Turkey and phenotypic data for future molecular studies of virulence traits of this important fish pathogen.

## 1 INTRODUCTION

Rainbow trout (*Oncorhynchus mykiss*) production has shown rapid development in the last 30 years in Turkey, and the total production volume reached 134,500 tons in 2021, making Turkey the largest portion size rainbow trout producer among European countries (FEAP, 2022). Sustainable fishery management depends on fish rearing in appropriate conditions and improving successful preventive health care (Li et al., 2011). In this respect, fish should be raised using good management practices, especially at the yolk sac stage and fry stage (Faruk & Anka, 2017). However, the intensive production of rainbow trout has resulted in increased health problems and disease‐related economic losses. Particularly, disease‐related mass mortalities in the early life stages of hatchery‐reared rainbow trout have been reported. Bacterial cold‐water disease (BCWD), also known as rainbow trout fry syndrome (RTFS), is a devastating infectious disease in freshwater salmonid farming that causes significant economic losses in hatcheries worldwide. The causative agent, *Flavobacterium psychrophilum*, is one of the most widely recognized pathogenic species of the *Flavobacterium* genus. BCWD outbreaks have frequently been reported in freshwater salmonid aquaculture worldwide and are associated not only with high mortality in rainbow trout but also in lake trout (*Salvelinus namaycush*) as well as Atlantic salmon (*Salmo salar*) (Bruce et al.,^2021^; Duchaud et al., 2018; Knupp et al., 2019).

*Flavobacterium psychrophilum* infections can result in high mortalities. Today, BCWD outbreaks in farmed fish are controlled by antibiotics administered through feed, which may lead to the dispersal of antibiotics in the environment with a risk of emergence of antibiotic resistance in *F. psychrophilum* and in the surrounding environmental bacteria (Bayliss et al., 2017; Gómez et al., 2014; Vliet et al., 2017).

Phylogenetic studies performed on *F. psychrophilum* strains isolated from diverse regions and fish hosts using multi‐locus sequence typing (MLST) revealed a strong association between genotypes and host species. In particular, the clonal complex CC‐ST10 represents the majority of the isolates associated to severe BCWD outbreaks in rainbow trout worldwide, and within, two sequences types (ST) are predominant in America (ST10) and European countries (ST2) (Avendaño‐Herrera et al., 2014; Knupp et al., 2019; Li et al., 2021; Nicolas et al., 2008; Nilsen et al., 2014; Van Vliet et al., 2016). The lack of efficient preventive measures and obstacles in developing protective vaccination strategies against *F. psychrophilum* (Gómez et al., 2014) may partly be due to the genetic and serological diversity within the species and to insufficient knowledge of the molecular pathogenesis. Virulence factors aid pathogenic bacteria to adhere to and invade the host, to evade host defence mechanisms and to cause disease (Maresso, 2019). Understanding molecular epidemiology and bacterial characteristics that contribute to virulence in a susceptible host is of crucial importance to develop efficient alternative control strategies (Bayliss et al., 2017).

*Flavobacterium psychrophilum* cells are highly proteolytic and adhesive (Bertolini et al., 1994; Papadopoulou et al., 2017). These phenotypic properties, mediated by secreted and surface proteins such as adhesins and proteases, rely on the activity of the Type IX secretion system (T9SS), a machinery found essential for pathogenicity in several *Bacteroidota*, such as the human pathogen *Porphyromonas gingivalis* and other animal pathogens such as *F. psychrophilum*, *F. columnare* and *Riemerella anatipestifer* (Barbier et al., 2020; Duchaud et al., 2007; N. Li et al., 2017; Paillat et al., 2023).

This study aims to determine the genomic characteristics (including serotype and MLST sequence type) as well as the in vitro phenotypic properties of *F. psychrophilum* Turkish isolates. This strategy should allow for a better knowledge of *F. psychrophilum* strains isolated in Turkish fish farms, and the data will help to initiate epidemiological investigations (including the monitoring of the propagation through the global trade of fish and eggs), detection of outbreak variants, and early warnings of emergence of epidemic strains that differ from the predominant clonal complex CC‐ST10. The identification of strains associated with severe BCWD outbreaks in Turkey, and a better knowledge of genetic determinants, will contribute to improve the accuracy of diagnosis, to develop control strategies, and to limit the unnecessary use of antibiotics in farms.

## 2 MATERIALS AND METHODS

### 2.1 Material

In the present study, we used 18 *F. psychrophilum* isolates recovered from disease outbreaks in fish farms in different geographical regions of Turkey including Aegean, Black Sea, Central Anatolia, East Anatolia and Marmara. Sixteen isolates were recovered from rainbow trout (*O. mykiss*), and 1 from Coruh trout (*Salmo coruhensis*) between 2014 and 2017 and were previously published in Saticioglu et al. (2018). Isolate FP‐U33, retrieved from a BCWD outbreak in rainbow trout in 2021, was also included. The information of isolates such as isolation source, fish species and isolation year is provided in Table 1.

**TABLE 1:** Information and in vitro phenotypic and genomic characteristics of *Flavobacterium psychrophilum* strains used in the study.

Isolate	Source			Hydrolysis			Abs@595	MLST data (WGS)			Type	Elastase encoding gene	Accession number
Code	Host	Region	Year	ELA	GEL	CAS	Adhesion	Allelic profile	ST	CC			
FP‐24	RT	C. A.	2014	+	+	+	1260 ± 0,006	2, 2, 2, 2, 2, 2, 2	ST2	CC‐ST10	2	FP061424_v1_150047	JAYKTD000000000
FP‐34	RT	Marmara	2015	−	+	+	1317 ± 0,044	2, 2, 2, 2, 2, 3, 41	ST92	CC‐ST10	2	Absent	JAYKTC000000000
FP‐37	RT	Black Sea	2015	+	+	+	0,951 ± 0,008	2, 2, 2, 2, 2, 2, 2	ST2	CC‐ST10	1	FP061426_v1_120036	JAYKTB000000000
FP‐73	RT	C. A.	2014	+	+	+	0,841 ± 0,153	2, 2, 2, 2, 2, 2, 2	ST2	CC‐ST10	2	FP061427_v1_150047	JAYKTA000000000
FP‐95	RT	Aegean	2014	+	+	+	1520 ± 0,021	2, 2, 2, 2, 2, 2, 2	ST2	CC‐ST10	1	FP061428_v1_160047	JAYKSZ000000000
FP‐100	RT	C. A.	2015	+	+	+	1446 ± 0,020	2, 2, 2, 2, 2, 2, 2	ST2	CC‐ST10	2	FP061429_v1_140048	JAYKSY000000000
FP‐108	RT	Aegean	2015	+	+	+	0,928 ± 0,035	2, 2, 2, 2, 2, 2, 2	ST10	CC‐ST10	1	FP061430_v1_160007	JAYKSX000000000
FP‐116	RT	Aegean	2014	W	+	+	1707 ± 0,029	2, 2, 2, 2, 2, 2, 2	ST2	CC‐ST10	1	FP061432_v1_160048	JAYKSW000000000
FP‐120	RT	Aegean	2015	+	+	+	1566 ± 0,035	2, 2, 2, 2, 2, 2, 2	ST2	CC‐ST10	1	FP120_v1_140007	JAYKSV000000000
FP‐123	RT	C.A.	2015	+	+	+	1314 ± 0,082	2, 2, 2, 2, 2, 2, 2	ST2	CC‐ST10	2	FP061434_v1_170047	JAYKSU000000000
FP‐311	RT	Aegean	2017	+	+	+	1165 ± 0,016	2, 2, 2, 8, 2, 2, 2	ST10	CC‐ST10	3	FP061435_v1_170048	JAYKST000000000
FP‐313	RT	Aegean	2017	+	+	+	1132 ± 0,009	2, 2, 2, 8, 2, 2, 2	ST10	CC‐ST10	3	FP313_v1_160007	JAYKSS000000000
FP‐314	RT	Aegean	2017	+	+	+	1014 ± 0,037	2, 2, 2, 8, 2, 2, 2	ST10	CC‐ST10	3	FP314_v1_150049	JAYKSR000000000
FP‐315	RT	Aegean	2017	+	+	+	1109 ± 0,035	2, 2, 2, 2, 2, 2, 2	ST2	CC‐ST10	2	FP061438_v1_150007	JAYKSQ000000000
FP‐335	RT	Aegean	2017	+	+	+	1384 ± 0,066	2, 2, 2, 2, 2, 2, 2	ST2	CC‐ST10	1	FP335_v1_160007	JAYKSP000000000
FP‐368	RT	C. A.	2017	+	+	+	0,637 ± 0,065	2, 2, 2, 2, 2, 2, 2	ST2	CC‐ST10	1	FP368_v1_170007	JAYKSO000000000
FP‐369	SC	Black Sea	2017	−	W	W	0,716 ± 0,019	12, 2, 5, 88, 3, 8, 3	ST393	n.d.	0	Absent	JAYKSN000000000
FP‐U33	RT	C. A.	2021	W	+	+	1232 ± 0,028	2, 2, 2, 2, 2, 2, 2	ST2	CC‐ST10	2	FP061442_v1_40007	JAYKSM000000000

Abbreviations: −, negative reaction; +, positive reaction; Allelic profile: *atpA*, *dnaK*, *fumC*, *gyrB*, *murG*, *trpB*, *tuf*; C.A., Central Anatolia; CAS, Casein; CC, clonal complex; ELA, Elastin; GEL, Gelatin; MLST, multilocus sequence typing; n.d., No data; RT, *Oncorhynchus mykiss*; SC, *Salmo coruhensis*; ST, sequence type; Typee: molecular serotypes inferred from *Type*genes encoding the different O‐antigen polymerases; W, Weak (i.e., clearing halo visualized only under the bacterial colony, no diffusible activity noticed).

### 2.2 Determination of in vitro phenotypic characteristics

Isolates were cultivated on Tryptone Yeast Extract Salts (TYES) agar supplemented with skimmed milk (5%), elastin (0.1%), gelatine (3%) for the determination of casein, elastin and gelatine hydrolysis, respectively, as described in Rochat et al. (2019) and Sundell et al. (2019). Broth suspension (1 μL) containing 10^9^ CFU mL^−1^ of each isolate was added to the agar surface in three replications. Petri dishes were incubated for 10 days at 15°C. Clearing zones formed around or under the colonies after incubation were recorded as positive. The adhesion measurement of *F. psychrophilum* was done following the methodology described by Högfors‐Rönnholm et al. (2015). Briefly, TYES broth cultures of *F. psychrophilum* isolates were diluted into lake water, sterilized by filtration (0.22 μm, MF‐Millipore), at a concentration of 10^9^ CFU mL^−1^. Post‐inoculation, aliquots of 100 μL were added in triplicate to a flat‐bottom 96‐well polystyrene microplate, using sterile lake water as the negative control. After 1 h of static incubation at 15°C, the supernatants were removed, and the wells were washed three times with sterile 0.5% NaCl to remove non‐adherent cells and allowed to dry at room temperature. Then, 125 μL of 0.1% crystal violet solution was added to each well and incubated for 45 min at room temperature. After the contents were discarded, the microplates were washed three times and dried. Then, 150 μL of 96% ethanol was added to each well and left at room temperature for 15 min. A 100 μL volume of solubilized crystal violet was transferred to a flat bottom microtitre plate and absorbance was measured in a microplate reader at 595 nm. The absorbance values were corrected by subtracting the average absorbance value of negative controls. The corrected values were presented as the mean (±standard deviation) of four biological replicates.

### 2.3 DNA extraction, genome sequencing and bioinformatic analyses

The genomic DNA of the 18 *F. psychrophilum* isolates was extracted using a QIAamp DNA mini kit (Qiagen, Hilden, Germany) following the manufacturer's instructions. Whole‐genome sequencing of isolates was performed on the Illumina HiSeq 2500 next‐generation sequencing platform with a 250‐bp paired‐end protocol by MicrobesNG (Birmingham, UK). The reads with low‐quality scores and adaptor contamination were trimmed using BBDuk, implemented in Geneious Prime. The high‐quality reads of the genomes were assembled into contigs by de novo assembly using the SPAdes assembler 3.13.0. The draft genome sequence data was submitted to the GenBank. The contigs longer than 1000 bp were annotated by the NCBI prokaryotic genome annotation pipeline (PGAP) (Tatusova et al., 2016).

Genomic similarity was estimated using the Mash software (Ondov et al., 2016) that computes a distance between two genomes. A tree was construct from all the pairwise distances of the genomes set using the neighbour‐joining javascript package V 1.0.4 (https://github.com/biosustain/neighbor‐joining). The core genome was defined using protein families built by sequences alignments of 70 available genomes and displaying at least 80% amino‐acids identity and 80% length coverage. Core‐genome tentative phylogenetic tree was constructed using 1487 single‐copy proteins. Protein alignments and concatenation were performed using in‐house scripts, Gblocks curation and FastTree reconstruction were performed using the NGPhylogeny.fr package with default parameters (Lemoine et al., 2019). The *wzy* gene types encoding various O‐antigen polymerases that determine the serotypes (Cisar et al., 2019; Rochat et al., 2017) were retrieved from genomic sequences. MLST sequence types (ST) were retrieved from PubMLST (https://pubmlst.org/) using genome assembly sequences as input (Nicolas et al., 2008).

## 3 RESULTS

### 3.1 Phylogenomic analysis

**FIGURE 1:**
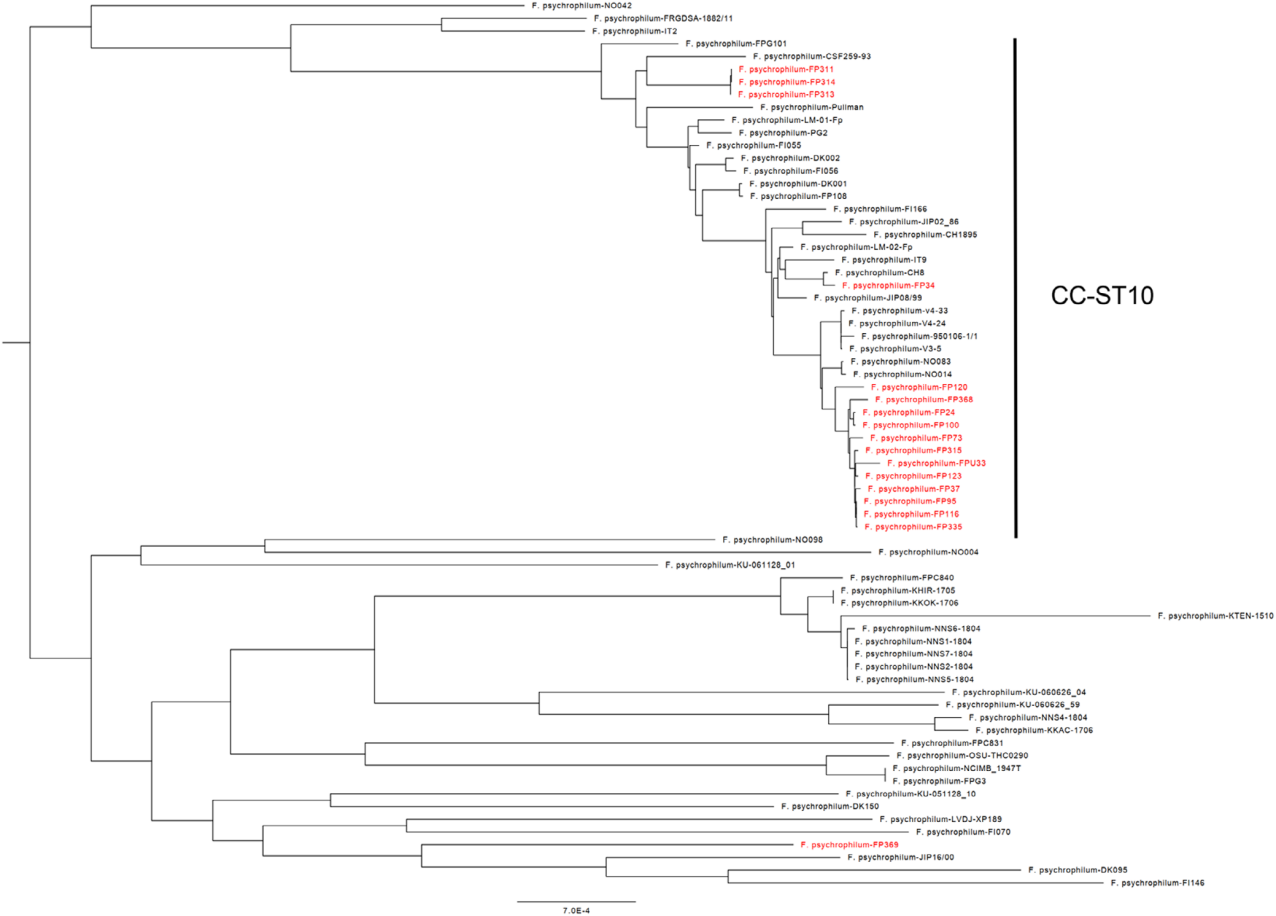
Core genome‐based tentative phylogenetic tree using 1487 single‐copy proteins retrieved from 18 Turkish isolates and previously published complete or draft genomes retrieved from Genbank.

Genome‐based tentative phylogenetic trees were constructed using nucleotide distances and core‐genome deduced proteins from the 18 Turkish isolates and previously published complete or draft genomes (Figure 1 and Figure S1). Turkish strains belonging to the CC‐ST10 are highly predominant (Table 1). Indeed, the 17 *F. psychrophilum* strains isolated from rainbow trout in fish farms of the Aegean, Black Sea, Central Anatolia, East Anatolia and Marmara regions all belong to this clonal complex. Within this group, sequence type ST2 is the most frequent (12 of 17). The only exception is strain FP‐369 which belongs to a previously unknown ST (ST393). Of importance, this strain was isolated from Coruh trout (*Salmo coruhensis*).

### 3.2 In vitro phenotypic characteristics

While the elastin hydrolysis of two isolates was negative, the elastin activity of the 16 other isolates was positive. Variations in the level of elastin activity were observed between isolates as determined by the appearance of the clearing halo which formed around the bacterial cells. The strains with the highest elastin hydrolysis (i.e., rapid appearance of a diffusible halo) are FP‐123 and FP‐315, whereas the activity is weak for FP‐U33 and FP‐116. Gelatine and casein hydrolysis of isolate FP‐369 is weak, while the other 17 isolates are positive. FP‐116, FP‐120 and FP‐95 have the highest adhesion activity. Results of in vitro proteolytic activity and adhesion assays of *F. psychrophilum* strains are summarized in Table 1.

### 3.3 Genomic characteristics

Draft genome sequences of strains were obtained and submitted to the NCBI GenBank database. The genome size of the strains is approximately 2.74 Mb ± 0.01 in average with GC content of 32.4% ± 0.1. Strain FP‐369 exhibits slightly higher genome length (2.95 Mb with GC content of 32.4%). Genome analysis allows for the identification of the O‐Ag encoding locus and the characterization of the *wzy* gene type which consequently allows for serotype inference (Table 1). An orthologue of the *F. psychrophilum* gene encoding the elastinolytic enzyme (FP0506 in reference strain JIP02/86), previously identified using a genome‐wide association study (Rochat et al., 2019), was present in the genome of all proficient elastin‐degrading cells identified in this study (Table 1). All genomes carry also the LRR encoding genes previously described (Duchaud et al., 2007; Wiens et al., 2014) and are suggested to be virulence factors in rainbow trout (Castillo et al., 2021).

## 4 DISCUSSION

The development of sustainable aquaculture requires an improved understanding of the circulating pathogens. The rapid development of the rainbow trout farming industry in Turkey raises significant sanitary issues and pathogen control now has now become of major concern. *F. psychrophilum* is the first cause of disease in Turkish rainbow trout farms especially at early live stages. In this study, we analysed an assortment of *F. psychrophilum* isolates recently obtained from BCWD outbreaks in Turkey (Saticioglu et al., 2018).

Early studies have shown *F. psychrophilum* to be actively proteolytic and proteases were predicted to be virulence factors of utmost importance leading to rapid and massive tissue destruction (Bertolini et al., 1994). In addition, genome mining and GWAS approaches predicted 14 secreted proteases (Duchaud et al., 2007; Rochat et al., 2019). Enzymatic capacities of *F. psychrophilum* cells to hydrolyse various protein substrates were performed. Only two isolates were deficient in elastin degradation, but all had gelatine and casein hydrolysis capacities. Important differences were observed between isolates for elastin degradation halo as previously observed for other strains in Rochat et al. (2019). This could be the consequence of many, and not mutually exclusive factors such as gene expression, protein secretion, cell lysis or the presence/abundance of extra cellular vesicles. Regarding adhesion ability, differences were noted among Turkish strains. Such phenotypic heterogeneity among *F. psychrophilum* isolates has been previously reported using polystyrene surface (Högfors‐Rönnholm et al., 2015) as well as gill arches (Nematollahi et al., 2003). Adhesion ability has been reported on fish surface (Kondo et al., 2002), mucus (Papadopoulou et al., 2017) and eggs (Vatsos et al., 2001). It has been suspected that adhesion is therefore important during the infectious process. However, no clear correlations have been yet reported between virulence in rainbow trout and haemolytic activity, in vitro adhesion, elastin, gelatine and casein hydrolysis properties (Sundell et al., 2019). Predicting virulence based on in vitro phenotypes prove to be poorly effective in *F. psychrophilum* except for avirulent isolates harbouring phenotypic deficiencies associated to loss of function of the T9SS (Jørgensen et al., 2022). Genomic analysis of these rainbow trout isolates by Castillo et al. (2021) further revealed no correlation between virulence and phylogeny as several phylogenetically closely related isolates harbour high or moderate virulence. Though, variations were observed between some isolates with contrasted virulence in the number of genes composing the LRR locus and of repeated domains of a putative adhesin, suggesting that these regions may be determinants of virulence in rainbow trout (Castillo et al., 2021). Indeed, bacterial LRR proteins were shown to mediate host–pathogen interactions and binding to host cell surface receptors (Loimaranta et al., 2009). Transcriptional induction of the LRR operon was shown in *F. psychrophilum* cells exposed to fish plasma, indicating their probable role in pathogenesis (Guérin et al., 2021). In the study herein, all genomes carry LRR genes but variations in the total number of duplicated copies and in the amino‐acid residues composition was not investigated due to scattering of the LRR locus in different contigs.

Genome‐based phylogenetic analysis and tentative phylogenetic tree reconstructions were performed using nucleotide distances and core‐genome deduced proteins (Figure 1 and Figure S1). Both trees were congruent with minor differences. These genomes were used to infer MLST‐type that revealed limited diversity among strains isolated from rainbow trout BCWD outbreaks. Strikingly, 12 of 17 rainbow trout strains are tightly connected in a single cluster (Figure 1). These 12 strains, all belonging to the ST2 genotype, were retrieved from rainbow trout farms located in different unrelated geographic locations (Central Anatolia, Marmara, Black Sea, Aegean). In contrast, no singleton ST from rainbow trout was identified suggesting that endemic isolates might occur rarely. The occurrence of genetically highly related strains in geographically distant areas (about 1000 km) and in different watersheds implies the dissemination at a nationwide scale of an epidemic rainbow trout‐associated bacterial population. The predominance of a limited number of rainbow trout‐associated genotypes (i.e., ST2 and ST10) has previously been reported in France (Siekoula‐Nguedia et al., 2012), in Switzerland (Strepparava et al., 2013) and in the Nordic countries (Nilsen et al., 2014), often in association with severe BCWD outbreaks. This finding is of particular epidemiological significance. It suggests that the Turkish fish farming practices (e.g., fish or eggs trade and movements), have favoured the expansion of a very few bacterial lineages.

The Turkish genomes were used to infer serotypes. Strikingly, FP‐369 is the only Type‐0 isolate. Type‐0, corresponding to Fp^T^ was mostly identified in *F. psychrophilum* isolates recovered from coho salmon (*Oncorhynchus kisutch*) (Rochat et al., 2017) but also frequent in *O. masou* in China (Li et al., 2021). On the contrary, Type‐0 are underrepresented in rainbow trout isolates and extremely rare in isolates from ayu (*Plecoglossus altivelis*). However, the mPCR serotyping scheme was originally defined to infers Types ‐1, ‐2 and ‐3 (Rochat et al., 2017) and consequently Type‐0 contains very heterogeneous strains. Indeed, recent comparisons of the O‐polysaccharide encoding loci suggested at least 17 different lipopolysaccharide serotypes in *F. psychrophilum* (Cisar et al., 2019). Therefore, further studies are needed to clarify lipopolysaccharide diversity among the Type‐0 strains. It should help to better understand the association between Type and host fish species, the ability of different serotypes to cause disease in a specific host and the ability of different serotypes to afford cross‐protection in vaccine studies. Among the CC‐ST10 strains, FP‐311, FP‐313 and FP‐314 are the only strains that harbour the *wzy3* gene encoding the O‐antigen polymerase associated to Type‐3 serotype. This serotype was largely predominant in strains isolated from ayu BCWD outbreaks, but very rare in rainbow trout associated isolates (Rochat et al., 2017). Structural differences in the O‐antigen may contribute to variation in the bacterial fitness depending of the fish host species (Izumi et al., 2003; Mata et al., 2002). However, all the others CC‐ST10 strains harbour the *wzy1* or *wzy2* gene reported to be rainbow trout associated (Rochat et al., 2017). Strikingly, the 12 tightly connected strains display intertwined Type 1 and Type 2 serotypes pointing out gene exchange by recombination at this locus. Consequently, it is tempting to speculate that recombination occurs during co‐infection in the same host by strains with unconnected serotype. Several authors reported co‐infection (or co‐existence) of *F. psychrophilum* in different fish host species (rainbow trout, ayu and coho salmon) in Japan (Fujiwara‐Nagata et al., 2013), Chile (Ilardi et al., 2023), France (Calvez et al., 2021) and the USA (Chen et al., 2008) some of which possessing different serotypes. It might be of interest to search such co‐infection events in future sampling campaigns.

Strain FP‐369 retrieved from *S. coruhensis* originates from a small local farm that produces various salmonid species. This situation is likely similar to the one described in Strepparava et al. (2013). This study also encompasses a unique strain (FP‐369) not retrieved from rainbow trout (namely, *S. coruhensis*). This strain belongs to a novel ST (namely, ST393) and is distantly connected to strain FI146 isolated from pond water in Finland, strain DK095 isolated from *Gasterosteus aculeatus* in Denmark and strain JIP16/00 isolated from *O. mykiss* in France (Figure 1). This strain should be considered as a singleton genotype. As previously reported, these singletons are usually recovered from a single or few fish host species and appeared to be geographically limited often associated with one fish farm (Calvez et al., 2021; Knupp et al., 2019; Van Vliet et al., 2016). This is likely the case for strain FP‐369. It could be of interest to assess the virulence of this strain in different hosts (i.e., *O. mykiss* and *S. coruhensis*) in order to explore bacterial genotype–host species interactions (Knupp et al., 2021). This strategy should help to reveal the role/importance of some molecular traits such as the presence/absence of the elastase (Rochat et al., 2019) or collagenase (Nakayama et al., 2016) encoding genes for host adaptation and/or virulence.

## 5 CONCLUSIONS

By combining in vitro phenotypic tests and genomic analysis we were able to observe at least to some extent heterogeneity among Turkish *F. psychrophilum* strains. These additional *F. psychrophilum* genome sequences should facilitate the understanding of virulence mechanisms in fish and provide a basis for the development of better control strategies. Ongoing genome sequencing and in vivo challenge tests of *F. psychrophilum* strains may help to provide new insights into pathogenicity and niche adaptation through comparative genomics. These results highlight the need for epidemiological surveillance of the isolates circulating in Turkey in order to adapt countermeasures, such as vaccines. In addition, they provide a cornerstone for future analyses of the virulence traits of this important fish pathogen.

## AUTHOR CONTRIBUTIONS

**Izzet Burcin Saticioglu:** Conceptualization; formal analysis; funding acquisition; investigation; project administration; writing – original draft; writing – review and editing. **Muhammed Duman:** Formal analysis; writing – original draft; writing – review and editing. **Nihed Ajmi:** Formal analysis. **Soner Altun:** Formal analysis; writing – review and editing. **Tatiana Rochat:** Investigation; writing – review and editing. **Eric Duchaud:** Conceptualization; formal analysis; writing – review and editing.

## CONFLICT OF INTEREST STATEMENT

The authors declare that they have no competing interests.

## Supporting information


Figure S1.

## Data Availability

Data presented within the manuscript are available in GenBank for draft genomes (see Table 1 for accession numbers) and in PubMLST for AT and ST of isolates.
