# Supplementary figures and images for: Phylogenomic characterization of Flavobacterium psychrophilum isolates retrieved from Turkish rainbow trout farms
Source: J Fish Dis. 2024 May 22;48(8):e13961. doi: 10.1111/jfd.13961 (PMC12285750; doi:10.1111/jfd.13961)

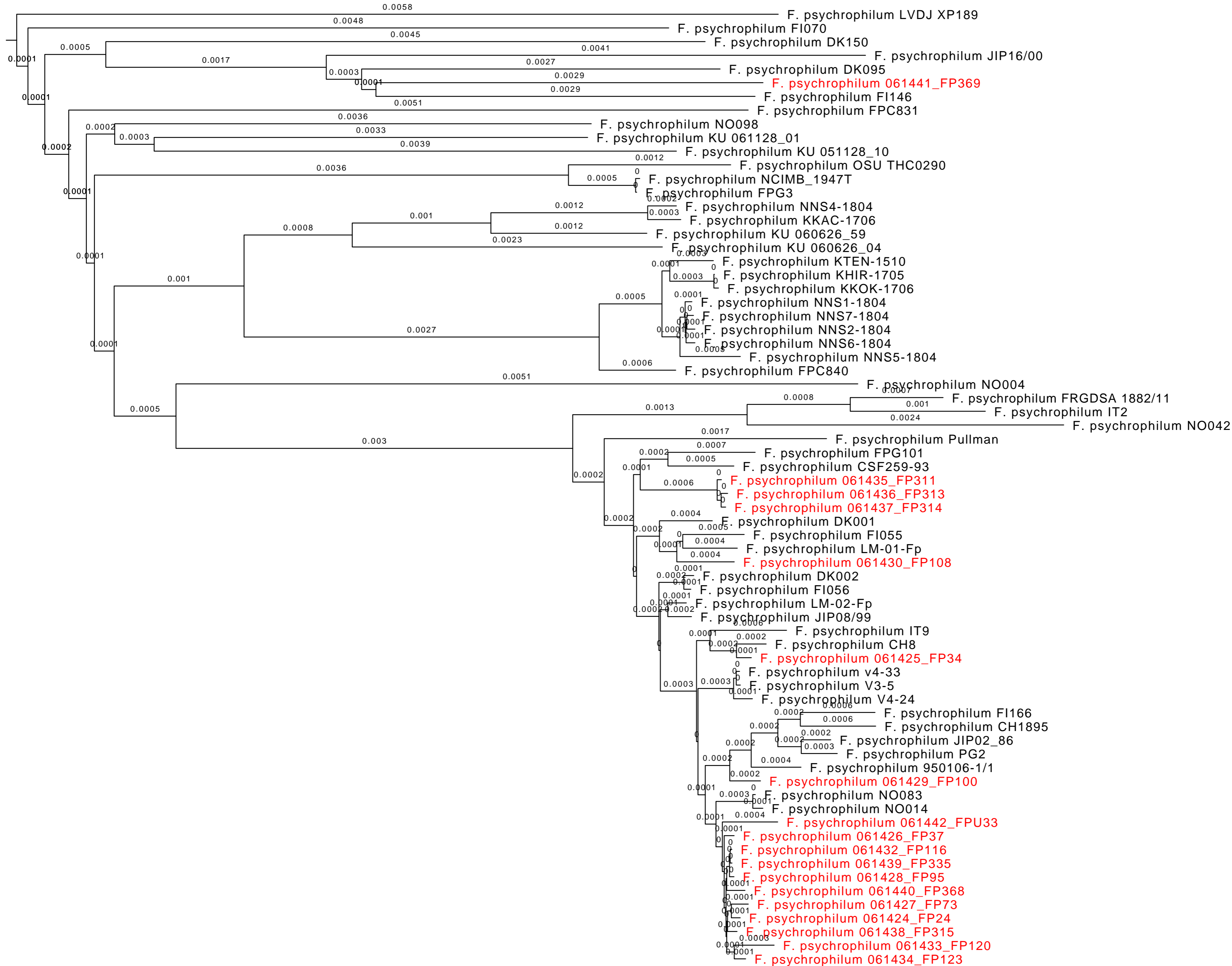

8.0E-4

Supplement: Supplementary file 1 — Figure S1. [file JFD-48-e13961-s001.pdf]
